# A Rare Cause of Short Stature: 3M Syndrome in a Patient with Novel Mutation in OBSL1 Gene

**DOI:** 10.4274/jcrpe.3238

**Published:** 2017-03-01

**Authors:** Melikşah Keskin, Nursel Muratoğlu Şahin, Erdal Kurnaz, Elvan Bayramoğlu, Şenay Savaş Erdeve, Zehra Aycan, Semra Çetinkaya

**Affiliations:** 1 Dr. Sami Ulus Obstetrics and Gynecology and Pediatrics Training and Research Hospital, Clinic of Pediatric Endocrinology, Ankara, Turkey

**Keywords:** childhood, short stature, genetic syndromes

## Abstract

The Miller-McKusick-Malvaux (3M) syndrome is a rare autosomal disorder that can lead to short stature, dysmorphic features, and skeletal abnormalities with normal intelligence. A 16-month-old female patient had been referred to our clinic due to short stature. Case history revealed a birth weight of 1740 grams on the 39^th^ week of gestation, with a birth length of 42 cm and no prior hereditary conditions of clinical significance in her family. On physical examination, her length was 67 cm [-3.6 standard deviation (SD) score], weight 7.2 kg (-2.9 SD score), and head circumference 42 cm (below 3^rd^ percentile). She also had numerous characteristic physical features such as a triangular face, fleshy nose tip, a long philtrum, prominent mouth and lips, pointed chin, lumbar lordosis, and prominent heels. As her growth retardation had a prenatal onset and the physical examination results were suggestive of a characteristic profile, the diagnosis of 3M syndrome was strongly considered. Genetic assessment of the patient revealed a novel homozygous p.T45Nfs*40 mutation in the *OBSL1* gene. It is recommended that physicians pay further attention to this condition in the differential diagnosis of children with severe short stature.

**WHAT IS ALREADY KNOWN ON THIS TOPIC?**

The 3M syndrome is a rare autosomal disorder that can lead to short stature, dysmorphic features, and skeletal abnormalities with normal intelligence levels. The 3M syndrome is caused by loss-of-function mutations in the genes encoding cullin 7, obscurin-like 1, and coiled-coil domain containing protein 8.

**WHAT THIS STUDY ADDS?**

The 3M syndrome may be a significant cause of short stature with prenatal onset in geographical regions where kin marriage is practiced extensively. Implementing frequent genetic screening for possible cases may help to identify novel mutations.

## INTRODUCTION

The Miller-McKusick-Malvaux (3M) syndrome is characterized by dysmorphic features, skeletal abnormalities, and severe prenatal as well as postnatal growth retardation with normal intelligence (1). The nomenclature 3M was derived from the initials of the surnames of three researchers who first identified the condition: namely, Miller, McKusick, and Malvaux (2). Although 3M syndrome is considered to be a relatively uncommon disorder, it is thought to possibly be an under-diagnosed condition (1).

The 3M syndrome is caused by loss-of-function mutations in the genes encoding cullin 7 (*CUL7*), obscurin-like 1 (*OBSL1*), and coiled-coil domain containing protein 8 (*CCDC8*). CUL7 appears to be the major gene responsible for 77% of 3M syndrome, while OBSL1 mutations account for a relatively small percentage of 16% (3). *CUL7* encodes for the *CUL7* protein that is a scaffold protein forming part of an E3 ubiquitin ligase enzyme responsible for cytoplasmic protein degradation. *OBSL1* encodes for a cytoskeletal adaptor protein which is localized within the prenuclear region. The function of *CCDC8*, on the other hand, is unknown. However, the protein it encodes for binds to OBSL1 protein and is required for p54-mediated apoptosis in cells. Detailed mechanisms underlying the growth impairments seen in the 3M syndrome remain largely unclear. On the other hand, abnormalities in basic cellular growth as well as alterations in cellular response profiles to growth factor stimulations are likely candidates for causal processes (1). There is no specific treatment for 3M syndrome (3). However, variable responses to growth hormone (GH) affected by the genotype, showing a better outcome in patients with *CCDC8* mutations in contrast to O*BSL1* mutations, have been reported too (4). Our patient, who demonstrated a previously unidentified mutation on *OBSL1* gene, was a one year and four months old female patient with a diagnosis of 3M.

## CASE REPORT

**Figure 1:**
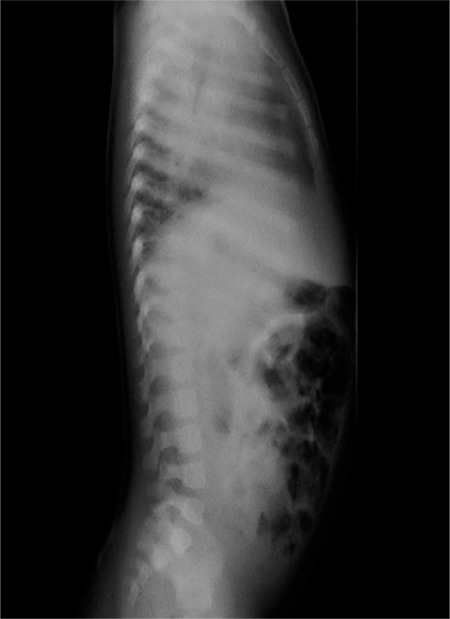
Radiography scan revealed mild reduction in thoracic vertebrae corpus height, with irregularities in upper and lower plateau as well as lordosis

A 16-month-old female patient was admitted to our clinic with a complaint of short stature. Her case history revealed that she was born as the first child of her consanguineous parents. The patient was born on the 39^th^ week of gestation with a birth weight of 1740 grams and birth length of 42 cm. There is no known prior case of hereditary disease in her family. On physical examination, the patient’s length was 67 cm [-3.6 standard deviation (SD) score], her body weight was 7.2 kg (-2.9 SD score), and head circumference 42 cm (below 3rd percentile). Her features consisted of a triangular face, a fleshy nose tip, a long philtrum, a prominent mouth and lips with a pointed chin alongside, lumbar lordosis, and prominent heels. Maternal body height was 164 cm and paternal height was 187 cm. Midparental height was estimated as 169 cm (+1 SD score). No noticeably short individual was reported in the family. The patient started forming syllables and walked approximately at one year of age. Laboratory analyses showed normal complete blood cell counts with normally functioning kidneys and liver. The patient was euthyroid and serological analyses for gluten-sensitive enteropathy were negative. Her bone age was in line with her chronological age. Her karyotype was 46,XX and bone X-rays revealed a lumbar lordosis (Figure 1). Echocardiographic findings were normal. Serum insulin-like growth factor 1 (IGF-1) level was 47 ng/mL (<-2 SD score) and serum IGF binding protein-3 (IGFBP-3) level was 2800 mg/mL (between +1/+2 SD score). L-dopa stimulation test scores revealed a peak GH response of 3.7 ng/mL.

**Figure 2:**
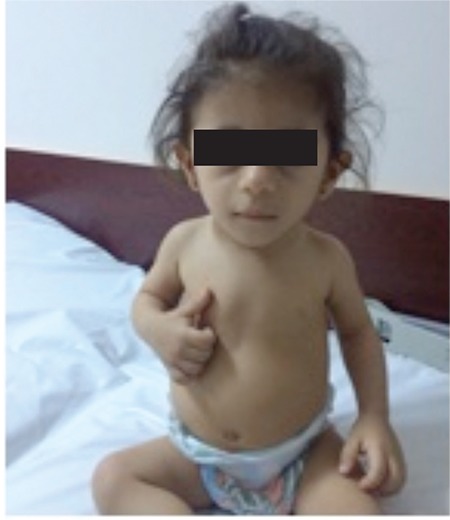
Our patient with her triangular face, long philtrum, fleshy nose tip, prominent mouth and lips and her pointed chin

Our patient had prenatal growth retardation and a significantly short stature in addition to triangular face, a long philtrum, a fleshy nose tip, prominent mouth and lips with pointed chin (Figure 2), as well as lumbar lordosis which collectively suggested a syndromic short stature. Drawing upon our prior experience with two sibling patients who were diagnosed with the 3M syndrome after a long period of undiagnosed clinical monitoring, we considered a 3M diagnosis to be appropriate for our current patient as well. The following genetic assessment, through the whole gene sequencing method, revealed a homozygous p.T45Nfs*40 (c.1273 dupA) mutation of *OBSL1* gene which led to diagnosis of 3M syndrome. Parental genetic analysis with same method also revealed that both of the parents had heterozygous mutations on *OBSL1*.

Clonidine stimulation test was planned as part of a secondary GH stimulation test. However, in view of the genetic profile of the patient (leading us to 3M diagnosis) and previous studies suggesting a degree of GH resistance as well as GH deficiency being possibly related with the 3M syndrome, and previous studies reporting that the 3M syndrome may be associated with the dysregulations of GH, IGF1, and IGF binding proteins, we refrained from applying the clonidine stimulation test to our patient (5). Hence, following the consent of her parents, a 0.25 mg/kg/week dose of GH was initiated. At the end of the first 3 months after treatment initialization, the patient showed a 4.5 cm growth, and this was followed by an additional 2.5 cm growth in the following three months period. On the 6th month of treatment, serum IGF-1 level was 272 ng/mL (+1/+2 SD score) and IGFBP-3 level was 4.87 µg/mL (+1/+2 SD score). The patient is currently under clinical observation and is being treated by a 0.25 mg/kg/week dose of GH.

## DISCUSSION

The 3M syndrome is a clinical condition which is often not diagnosed during childhood (2). Here, we describe a 16-month-old female patient with the diagnosis of 3M syndrome. This individual applied to our clinic due to complaints of pre- and post-natal growth retardation. 3M diagnosis was considered following the clinical assessment for short stature. However, the 3M syndrome can appear with mild symptomatology and is a difficult condition to identify via differential diagnosis as short stature is known to have a wide variety of causal factors (3). We consider this to be the main reason for poor numbers of 3M diagnoses and significantly delayed diagnoses for those with the syndrome (5). This is a situation that can be harmful for prognosis, as an early diagnosis is crucial for genetic counselling since 3M syndrome is inherited as an autosomal recessive disorder (2).

The 3M syndrome is causally linked with the mutations on the genes *CUL7, OBSL1*, and *CCDC8*. A previous study showed that patients who were diagnosed with the 3M syndrome, having mutations on these genes, tended to be shorter to the degree of a -5.7 SD score for *CUL7*, a -4.7 SD score for OBSL1, and a -4.1 SD score for *CCDC8* (6). OBSL1 gene mutations are the underlying causes for approximately 20% of the 3M syndrome patients (3). Mutation types reported include insertion, deletion, and substitution of nucleotides, all appearing on the first eight exons encoding for Ig domains of *OBSL1* proteins (7). The c.1273insA (p.T245fs*40) mutation had been identified as the prevalent mutation for the *OBSL1* gene in 12 of 23 families that had undergone screening (8). In our case, on the other hand, we observed that a novel frameshift mutation on *OBSL1* caused the 3M syndrome. Currently, little is known about the specific functions of *OBSL1*; yet, it was suggested that the *OBSL1* protein functions as a cytoskeletal adaptor protein linking the nuclear proteins to the cytoplasmic support network. Additionally, *OBSL1* was also found to be expressed in a wide variety of cell types, suggestive of its role as a scaffolding protein (9). In addition, alterations in IGFBP-2 and IGFB5 messenger ribonucleic acid levels were previously documented to be associated with *OBSL1* mutations in cases with 3M syndrome diagnoses (7).

There is no specific treatment for 3M syndrome (3). However, the use of recombinant human GH for the treatment of short stature was suggested (7). Previous studies suggested a degree of GH resistance as well as GH deficiency being possibly related with the 3M syndrome, and it was also reported that the 3M syndrome may be associated with the dysregulations of GH, IGF1, and IGF binding proteins (5). Significant individual variations were also reported in relation to GH responses and some studies also suggest that GH may be helpful in the treatment of the syndrome (10). On the other hand, according to various other reports, GH treatments have no effect on patients with the 3M syndrome (7,11). Even though GH treatment outcomes for the 3M syndrome appear controversial, we decided to initiate GH treatment since the expected final height of our patient appeared to be relatively short in view of previous literature about this syndrome. At the end of the first six months post-initiation with a 0.25 mg/kg/week dose, our patient demonstrated a 7-cm growth increment. Even though the duration of clinical observation was inadequate, the finding of a sufficient rate in growth for that specific duration was satisfactory in deciding on a close clinical observation of serum IGF-1 and IGFBP-3 levels for the remaining duration of treatment.

The 3M syndrome may be more frequent than thought in countries such as Turkey, where kin marriage is a frequent practice. Pointing out this fact may help clinicians working in Turkey or other countries with similar practices to consider this syndrome in the diagnostic work-up of their future patients, hence revealing increasing numbers of cases in the near future. Our experience with previous 3M diagnoses is an example to this fact, as our case, which can be identified as a case of early diagnosis, could have been diagnosed mainly with respect to our prior knowledge regarding 3M syndrome. It was previously reported that final heights of the 3M syndrome patients range between 115 and 150 cm, which can lead to significant degrees of disadvantage in these individuals’ lives (4). Since our patient is of a very young age, we postulate on the possibility that the initiation of GH treatment can, with a high chance, lead to a near-normal body height. Due to the fact that 3M syndrome is inherited via an autosomal recessive pattern, early genetic assessment leading to an early diagnosis can also aid in the genetic counselling for the rest of family members. In conclusion, 3M syndrome needs to be considered in the differential diagnosis of patients with growth failure, especially those with prenatal onset and characteristic symptoms.
